# What is the value and impact of the adaptation process on quality indicators for local use? A scoping review

**DOI:** 10.1371/journal.pone.0278379

**Published:** 2022-12-08

**Authors:** Siyi Zhu, Tao Wu, Jenny Leese, Linda C. Li, Chengqi He, Lin Yang

**Affiliations:** 1 Rehabilitation Medicine Center, West China Hospital, Sichuan University, Chengdu, China; 2 Rehabilitation Key Laboratory of Sichuan Province, West China Hospital, Sichuan University, Chengdu, China; 3 Arthritis Research Canada, Vancouver, British Columbia, Canada; 4 Faculty of Medicine, School of Epidemiology and Public Health, University of Ottawa, Ottawa, Ontario, Canada; 5 Department of Physical Therapy, University of British Columbia, Vancouver, British Columbia, Canada; University of Florida, UNITED STATES

## Abstract

**Background:**

Quality indicators (QIs) are designed for improving quality of care, but the development of QIs is resource intensive and time consuming.

**Objective:**

To describe and identify the impact and potential attributes of the adaptation process for the local use of existing QIs.

**Data sources:**

EMBASE, MEDLINE, CINAHL and grey literature were searched.

**Study selection:**

Literatures operationalizing or implementing QIs that were developed in a different jurisdiction from the place where the QIs were included.

**Results:**

Of 7704 citations identified, 10 out of 33 articles were included. Our results revealed a lack of definition and conceptualization for an adaptation process in which an existing set of QIs was applied. Four out of ten studies involved a consensus process (e.g., Delphi or RAND process) to determine the suitability of QIs for local use. QIs for chronic conditions in primary and secondary settings were mostly used for adaptation. Of the ones that underwent a consensus process, 56.3 to 85.7% of original QIs were considered valid for local use, and 2 to 21.8% of proposed QIs were newly added. Four attributes should be considered in the adaptation: 1) identifying areas/conditions; 2) a consensus process; 3) proposing adapted QIs; 4) operationalization and evaluation.

**Conclusion:**

The existing QIs, although serving as a good starting point, were not adequately adapted before for use in a different jurisdiction from their origin. Adaptation of QIs under a systematic approach is critical for informing future research planning for QIs adaptation and potentially establishing a new pathway for healthcare improvement.

## Introduction

Quality improvement is a cornerstone in health care and clinical practice, and Institute of Medicine defined quality of care as “the degree to which health services for individuals and populations increase the likelihood of desired health outcomes and are consistent with current professional knowledge” [1]. Performance measurement is one of the five domains involved in improving quality of care [2]. However, clinical uncertainty about practice and quality of care varies remarkably in reality. For example, divergence from evidence-based care is commonly seen in care for osteoarthritis and other conditions, where only around half of patients receive the guideline-recommended treatment [3]. Therefore, tremendous efforts have been made to investigate how and for which conditions quality deficiencies stand so as to target where improvements are needed. It is increasingly common for countries around the world to set national benchmarking and public standards to measure or monitor the quality of care by means of indicators [4].

Quality indicators (QIs) are “standardized, evidence-based measures of healthcare quality that can be used to measure and track health status and health system performance, and characteristics across different populations, between jurisdictions, or over time” [5]. Researchers in the RAND group developed a consensus method (similar to the development of clinical guidelines) for systematically combining best evidence with expert opinion to assess the appropriateness of healthcare and lead the way in the use of QIs [6]. With using a modified version of this method, QI statements specifying context structure, care process and expected outcomes are generated using the synthesized evidence or clinical guideline recommendations [7]. Typically, QIs include statements about the structure, process and outcomes of care in the ‘IF-THEN-BECAUSE’ format [8, 9]. The “IF” statement determines eligibility for the care process in question, the “THEN” statement specifies what care process should be performed, and the “BECAUSE” touches on the expected health impact where the indicator is implemented [10]. An example of a quality indicator is as follows: “IF a patient has symptomatic osteoarthritis of the knee or hip and has been overweight for 3 years THEN he/she should receive referral to a weight loss program” [11]. On different levels and for various stakeholders, QIs have been developed and applied to facilitate regulation, measure performance, advance accountability and improve the quality of care. Policymakers can use QIs as a tool to track and evaluate the impact of policies. Public and private payers are interested in QIs to measure the cost-effectiveness of treatment prior to approving payment in a manner of pay-for-performance schemes. Health professionals can use QIs to intensively monitor the practice and improve the performance accordingly [12]. QIs are an important source of consumer info for patients considering any treatment choice. For patients like with osteoarthritis, QIs were developed in 2004 by the Arthritis Foundation, which represent the minimal and evidence-based care that should be received by the targeted population [11].

Large-scale efforts to develop and implement QIs for healthcare in many conditions have been exerted mostly in the US and Europe [5, 13–15]. However, the development process of QIs is time-consuming and comes at a high cost, so it is reasonable for countries to use existing QIs rather than develop their own. In the US, four years between 1995 and 1999 were taken to develop the RAND QIs, while researchers in the UK adapted this set of QIs for local use in less than two years [16]. Transferring QIs between countries with an intermediate process is therefore feasible, but the rate of agreement on accepting QIs for local use differed remarkably. Care of the elderly is considered as an increasingly important component in healthcare service worldwide. The Assessing Care Of Vulnerable Elderly (ACOVE) QIs were developed aiming to evaluate and optimize the care for elderly patients in US by Rand Healthcare and the UCLA [17]. Several studies adapted ACOVE QIs for local use in the UK and Netherlands with an approval rate (# of original QIs to # of proposed QIs in the adaptation) varying from 56–86% [16, 18, 19]. Further, there also have been examples in which QIs developed in one jurisdiction were used in another region without adaptation under a direct-adoption process [20, 21]. An expert panel from England rated 79 of 93 chosen ACOVE QIs as valid for local use without any amendment [18]. Although there are potential benefits in using an existing set of QIs as a starting point to adapt a set of QIs for local use, it is unclear what an adaptation process should involve transferring a set of QIs for local use, and whether such a process is always appropriate or desirable in adapting or applying a set of QIs. It is worth noting that a cross-cultural adaptation of a quality measurement should involve the development of versions equivalent to the original QIs, however simultaneously linguistically and culturally adjusted to the local context, in which the first step is to conduct a scoping review to understand the state-of-the art regarding the QIs selection and development [22]. Therefore, the aim of this study was to review the literature on the process used for adapting an existing set of QI’s for local use.

## Materials and methods

A scoping review protocol was guided by the work of Arksey and O’Malley [23] and further refined by Peters Micah et al. initiated by the Joanna Briggs Institute [24]. The draft protocol was revised based on feedback received from co-authors and registered in an international prospective registry of systematic reviews (PROSPERO; reference number: CRD42018096844) [25]. The Preferred Reporting Items for Systematic Reviews and Meta-Analyses extension for Scoping Reviews (PRISMA-ScR) guidelines were followed [26].

For the purpose of the review, a quality indicator was defined as a measure transformed into a statement that was validated and presented as percentage or proportion describing quality of care delivered [27]. We defined an adaptation process as a standardized process that purposefully selected a set of existing QIs (with or without an intermediate process, e.g., RAND or Delphi process) and transformed them into a set that was deemed suitable for use in the local context, before a pilot testing or full-scale implementation.

### Search strategy

Search strategies were developed combining terms of subject headings and text words related to concepts of QIs and adaptation processes (Supplementary materials). EMBASE, MEDLINE and CINAHL were searched from January 1990 to September 2019. A search of Google Scholar using keywords including “quality indicators”, “quality improvement”, “healthcare”, “cross-cultural adaptation”, “transferability”, and websites of quality improvement research were conducted to identify grey literature. A librarian specializing in medical information helped revise searches. The reference lists of included studies were reviewed to identify additional eligible studies. An additional search was performed under a mechanism of living systematic review [28] to identify recently published relevant studies from October 2019 to October 2022 using the databases and keywords described above.

### Study selection

Endnote X9 was used to manage citations retrieved from searches and remove duplicates. Studies were eligible if they 1) adapted or operationalized a set of existing QIs for local use; 2) assessed the acceptability or usability of adapted or operationalized QIs, with a definition of to what extent the indicator was judged as acceptable or practical for local use by those being assessed or those performing the assessment; 3) published in English or Chinese. Two reviewers independently conducted the level 1 (titles and abstracts) and level 2 (full text) screenings. A screening tool was developed and tested on a sample of citations retrieved prior to the full review (Supplementary materials). Any disagreement between the two was discussed and, if needed, resolved through discussion with a third party.

### Data collection and synthesis

For included studies, data were extracted on study characteristics (e.g., first author, year and country of publication), participants/population description, healthcare settings, process involved in adaptation, objectives and key conclusions. QIs were classified as the original set and adapted set based on the aspects of structure, process and outcome assessed. Information on QIs used and adapted, test results where applicable and any finding related to the adaptation were reviewed and mapped. A data extraction form was developed and piloted on a sample of citations prior to the data extraction. Two reviewers were involved independently, and consensus was used to resolve any disagreements, or arbitration by a third party if applicable.

### Quality assessment

The quality of included studies was assessed by two reviewers using Revised Standards for Quality Improvement Reporting Excellence (SQUIRE 2.0), a framework with 18 items for reporting new knowledge about how to improve healthcare [29]. A quality score out of eighteen was given to all studies included. Two reviewers scored studies independently, and agreements were achieved by either consensus or arbitration by a third party if applicable.

## Results

**Fig 1:**
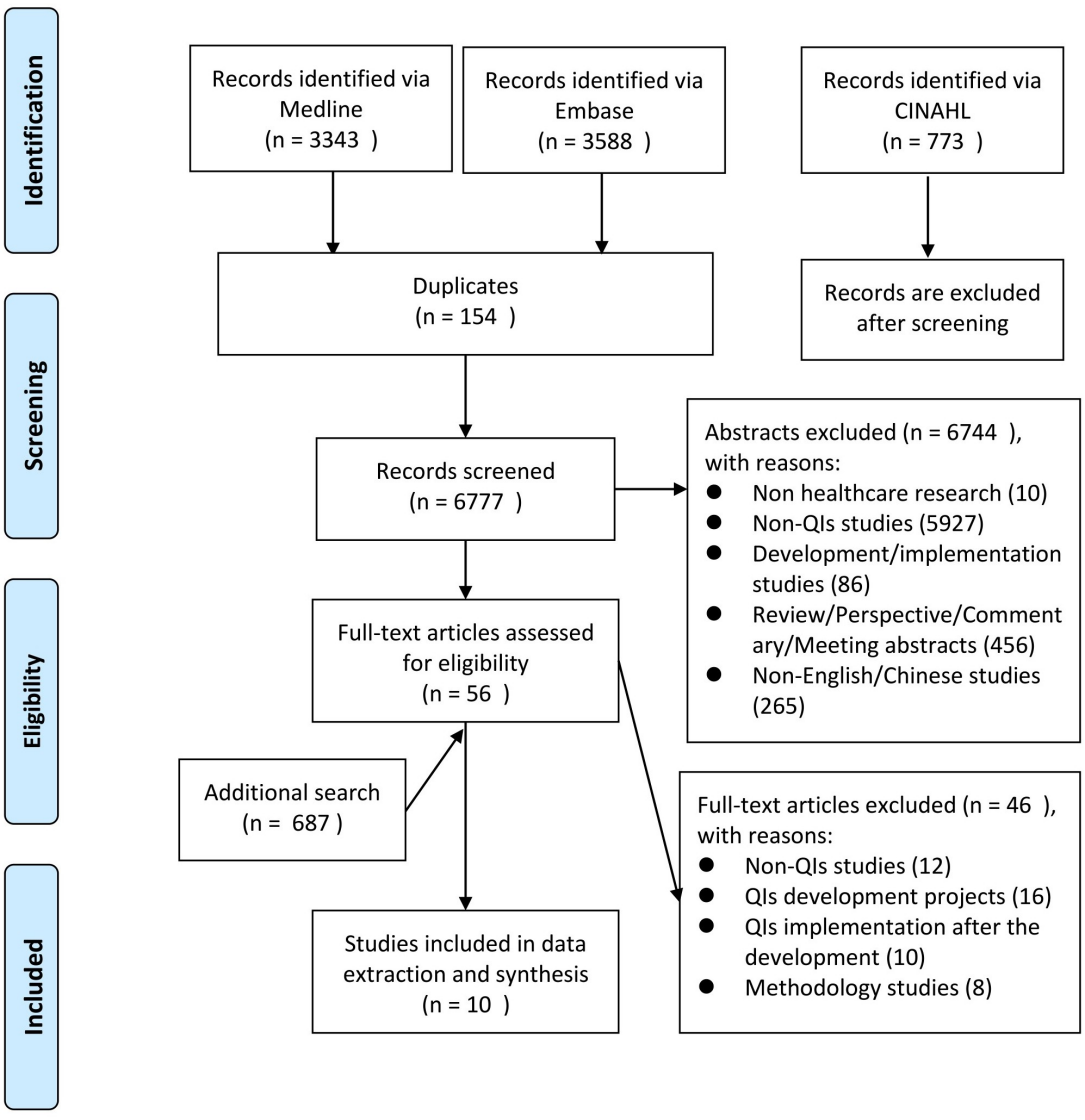
Flow diagram for study inclusion.

The search yielded 7704 articles plus 687 records retrieved from the additional search and grey literatures, of which 56 articles were identified eligible for full-text screening after the removal of duplicates and review of titles and abstracts. Of these, 10 articles [16, 18–21, 30–34] were included in this study for data extraction and synthesis, 6 of which were identified manually by authors (Fig 1). For 90% of included studies, the primary aim was to assess acceptability of QIs transferred from one country to another with an intermediate or direct-adoption process, and one study [32] was conducted to assess quality of care by operationalizing QIs locally (Table 2). Studies were excluded because they did not involve adapting or operationalizing a set of existing QIs for local use. The main reasons for exclusion were no QI used in the study (n = 9) or no adaptation process was described (n = 20). Of the latter, 10 were QIs development studies, eight were QIs implementation studies, and two were methodology studies.

### Quality of included studies

Articles included were assessed as high-quality, with SQUIRE 2.0 scores [29] of 18/18 for 4 studies, 17/18 for 3 studies, 16/18 for 2 studies and 15/18 for one study. Ethical consideration, the description of limitation and indicating funding resources were identified as three main weaknesses in the quality assessment. The findings are summarized in Table 1.

**Table 1:** Summary of the SQUIRE statement quality appraisal results criterion.

Study	Criterion*																	
	1	2	3	4	5	6	7	8	9	10	11	12	13	14	15	16	17	18
Effendy 2014 [30]	1	1	1	1	1	1	1	1	1	1	1	0	1	1	1	1	1	0
Steel 2004 [18]	1	1	1	1	1	1	1	1	1	0	1	0	1	1	1	0	1	1
van der Ploeg 2008 [19]	1	1	1	1	1	1	1	1	1	1	1	1	1	1	1	1	1	1
Marshall 2003 [16]	1	1	1	1	1	1	1	1	1	1	1	0	1	1	1	0	1	1
Jeon 2017 [21]	1	1	1	1	1	1	1	1	1	1	1	1	1	1	1	1	1	1
Pellesi 2017 [33]	1	1	1	1	1	1	1	1	1	1	1	1	1	1	1	1	1	1
Schramm 2016 [34]	1	1	1	1	1	1	1	1	1	1	1	1	1	1	1	1	1	1
Hansen 2013 [20]	1	1	1	1	1	1	1	1	1	1	1	0	1	1	1	1	1	1
Katsarava 2015 [31]	1	1	1	1	1	1	1	1	1	1	1	1	1	1	1	0	1	1
Li 2011 [32]	1	1	1	1	1	1	1	1	1	1	1	1	1	1	1	1	1	0

*Revised Standards for Quality Improvement Reporting Excellence (SQUIRE 2.0): 1. Title; 2. Abstract; 3. Problem Description; 4. Available knowledge; 5. Rationale; 6. Specific aims; 7. Context; 8. Intervention(s); 9. Study of the Intervention(s); 10. Measures; 11. Analysis; 12. Ethical Considerations; 13. Results; 14. Summary; 15. Interpretation; 16. Limitations; 17. Conclusions; 18. Funding.

^†^Notes: 0 = no, 1 = yes.

### Study characteristics

Table 2 summarizes the characteristics of studies included in the study. The years of publication ranged from 2002 to 2017. Seven out of 10 studies were undertaken in Europe including the UK (2), European consortium (2), Netherlands (1), Denmark (1) and Italy (1). Two studies [21, 30] were from Asia-Pacific regions and one was from Canada. The participants involved in included studies varied with respect to clinical disciplines and how QIs were adapted. These disciplines included geriatric medicine (3), headache conditions (3), palliative care (1), mental health (1), respiratory medicine (1) and rheumatology (1). Four studies [16, 18, 19, 30] formed a multi-disciplinary expert panel to assess the transferability of QIs between countries through an intermediate process supported by the RAND/UCLA and Delphi methods. Other studies tested the QIs in at least one target population in which the QIs were originally developed. These included health care professionals, health services managers, and patients. The acceptability of QIs was also assessed by reviewing medical records or results of surveys in the latter group of studies included. For the setting where QIs were adapted, six studies were conducted in the general practice setting or primary and secondary settings, others were conducted in the secondary or tertiary setting.

**Table 2:** Study characteristics.

First author (Year)	Country of publication	Population/participants description	Settings	Steps involved in employing quality indicators	Aim of the study	Key Conclusion
Effendy 2014 [30]	Indonesia	12 experts in palliative care or cancer care and 12 additional experts involved in a multidisciplinary panel.	Palliative care in hospital setting	Modified RAND Delphi process	1) Face-validate an Indonesian set of QIs based on a European set; 2) Compare and test the applicability of Indonesian set.	Most of the European set were face-valid and applicable in Indonesian hospitals.
Steel 2004 [18]	UK	An expert panel consisted of 6 consultant physicians in geriatric medicine or medicine for the elderly, 3 general practitioners, and 1 nurse.	Primary and secondary care setting	RAND/UCLA appropriateness method	1) Adapt a set of USA quality indicators in England; 2) Measure the extent to which older patients receive a broad range of effective healthcare interventions.	These 102 indicators are suitable for use in patient interview surveys in England, and there is potential for transfer of quality indicators between countries.
van der Ploeg 2008 [19]	Netherlands	A panel consisted of 8 general practitioners, 2 nursing-home practitioners and 2 clinical geriatricians.	General practice care	RAND/UCLA process	1) Adapt a set of USA quality indicators in Netherlands; 2) Test the transferability of quality indicators between countries.	Quality indicators can be transferred between countries but with caution.
Marshall 2003 [16]	UK	Two panels of UK general practitioners were recruited from 196 doctors, of which 75% agreed to take part. Nine members for each of two panels were selected purposely.	General practice care	RAND/UCLA process	Evaluate the transferability of primary care quality indicators developed using the same method between the UK and the USA.	Considerable benefits using work from other settings are presented for developing measures of quality of care, but indicators should not be transferred between countries without adaptation.
Jeon 2017 [21]	7 Asia-Pacific countries	Member country representatives of the PROMOTE (Psychosocial Research Consortium to Advance Mental Health of Older People in the Asia-Pacific region). Medical records of 275 residents with dementia were extracted.	Institutional or long-term care setting	Not applicable	Determine whether or not European quality indicators (QIs) for psychosocial care in dementia could be implemented across seven Asia-Pacific countries.	Several indicators in the European set were problematic with the direct adoption in Asia-Pacific region, and refinements of the original set are required in future.
Pellesi 2017 [33]	Italy	Six headache specialist centres were recruited via a network of young Italian researchers on headache. Service records from the physicians and other HCPs (nurses, psychologists and/or nursing assistants) involved in outpatient consultations, and from 60 consecutive patients were extracted.	Headache specialist centres (level 3)	Not applicable	Extend implementation of quality indicators and examine acceptability and ease of use of the assessment instrument in Italian specialist headache centres.	This Italy-wide survey confirmed in six specialist centres that the headache service quality indicators are fit for purpose.
Schramm 2016 [34]	12 countries in Europe	Service staff and patients were study participants. The patient participants at each centre were a prospective consecutive sample (n = 50). In addition, information was acquired from the records of a retrospective random or consecutive sample of 50 patients.	Secondary (level-2) or tertiary (level-3) headache clinics	Not applicable	Evaluate the implementation of quality indicators for headache care Europe-wide in specialist headache centres	The quality indicators were workable in specialist care across Europe.
Hansen 2013 [20]	Denmark	102 Danish general practitioners accepted to participate.	General Practice care	Not applicable	Assess a set of 41 newly developed international quality indicators for antibiotic treatment of respiratory tract infections.	Only a few of the set of quality indicators developed by international panel were rated suitable by the GPs.
Katsarava 2015 [31]	Germany and Portugal	In each centre the health professionals most involved in delivering the service were interviewed, and consecutive patients and their records were investigated. Numbers of patients at each centre were determined by what was feasible.	Primary and secondary headache clinics	Not applicable	Evaluate the feasibility and acceptability of quality indicators in the two countries.	This pilot study to assess feasibility of the methods and acceptability of the instruments of headache service quality evaluation was successful.
Li 2011 [32]	Canada	Patients with hip and/or knee osteoarthritis (OA) who participated in a population-based survey (n = 1,349). They were from a random sample of 6000 patients identified using administrative databases collected by the Ministry of Health of British Columbia, for the purpose of reimbursements for outpatient physician visits and hospitalizations.	Outpatient and inpatient setting	Not applicable	To assess the quality of nonpharmacological care received by people with knee and/or hip osteoarthritis (OA) in the community and to assess the associated factors.	The quality of nonpharmacological care for people with knee/hip OA in the community is suboptimal.

### Adaptation of quality indicators

Table 3 maps the use of existing QIs, with or without adaptation. Seven separate sets of original QIs were employed in the included studies, of which three were from the USA, two were from Europe, and two were developed by an international consortium. Of these existing QIs targeted, the process domain was covered in all studies, the structure domain was addressed in four studies, while one study addressed the outcome domain.

**Table 3:** Adaptation of Quality Indicators (QIs).

Ref	Original set of QIs	Country of the original set	Country of the adaptation	QIs Classification	QIs adapted (N)							Other findings related to the adaptation and implementation
					Proposed in total	Original set	Newly added	Valid	Significantly changed	Discarded	Approval rate of proposed QIs	
Effendy 2014 [30]	A set of QIs for the organization of palliative care, previously developed in the European Europall project	Seven European countries	Indonesia	Structure, Process	100	98	2	78	N/A	22	78.0%	N/A
Steel 2004 [18]	Assessing the Care of Vulnerable Elders (ACOVE)	USA	UK	Process	119	93	26	102	N/A	17	85.7%	N/A
van der Ploeg 2008 [19]	ACOVE-3	USA	Netherlands	Process	113	108	5	77	4	32	68.1%	N/A
Marshall 2003 [16]	The RAND quality indicators (termed the “QA tools”)	USA	UK	Process	174	174	N/A	98	N/A	76	56.3%	N/A
Jeon 2017 [21]	A set of 12 QIs developed by a multinational European consortium	Europe	Asia-Pacific region	Process	12	12	N/A	N/A	N/A	N/A	N/A	QIs were not fully acceptable and auditors had difficulty in scoring 7 of 12 QIs.
Pellesi 2017 [33]	A set of 30 QIs developed by Lifting the Burden (LTB)	UK/WHO	Italy	Structure, Process and Outcome	30	30	N/A	N/A	N/A	N/A	N/A	QIs were reported as easy to apply, readily understood and not unduly time consuming.
Schramm 2016 [34]	A set of 30 QIs developed by Lifting the Burden (LTB)	UK/WHO	Europe	Structure, Process and Outcome	30	30	N/A	N/A	N/A	N/A	N/A	QIs in 10 different language were reported as easy to apply and understood, no difficulty of use was found.
Hansen 2013 [20]	A set of 41 QIs developed by a international consortium	International	Denmark	Process	41	41	N/A	N/A	N/A	N/A	N/A	None of the QIs were assessed by all 58 GPs to be suitable as a good assessment tool. 33 Danish GPs indicated that more than 50% of the QIs were suitable, and 25 GPs agreed on less than 50% of the 41 QIs.
Katsarava 2015 [31]	A set of 30 QIs developed by Lifting the Burden (LTB)	UK/WHO	Germany and Portugal	Structure, Process and Outcome	30	30	N/A	N/A	N/A	N/A	N/A	QIs proved easy to apply, were readily understood and accepted by both health professionals and patients and were not unduly time consuming.
Li 2011 [32]	Arthritis Foundation QIs for Osteoarthritis (OA)	USA	Canada	Process	4	14	N/A	N/A	N/A	N/A	N/A	QIs were used in the OA population from British Columbia Canada without modification.

*N/A: Not Applicable.

**Fig 2:**
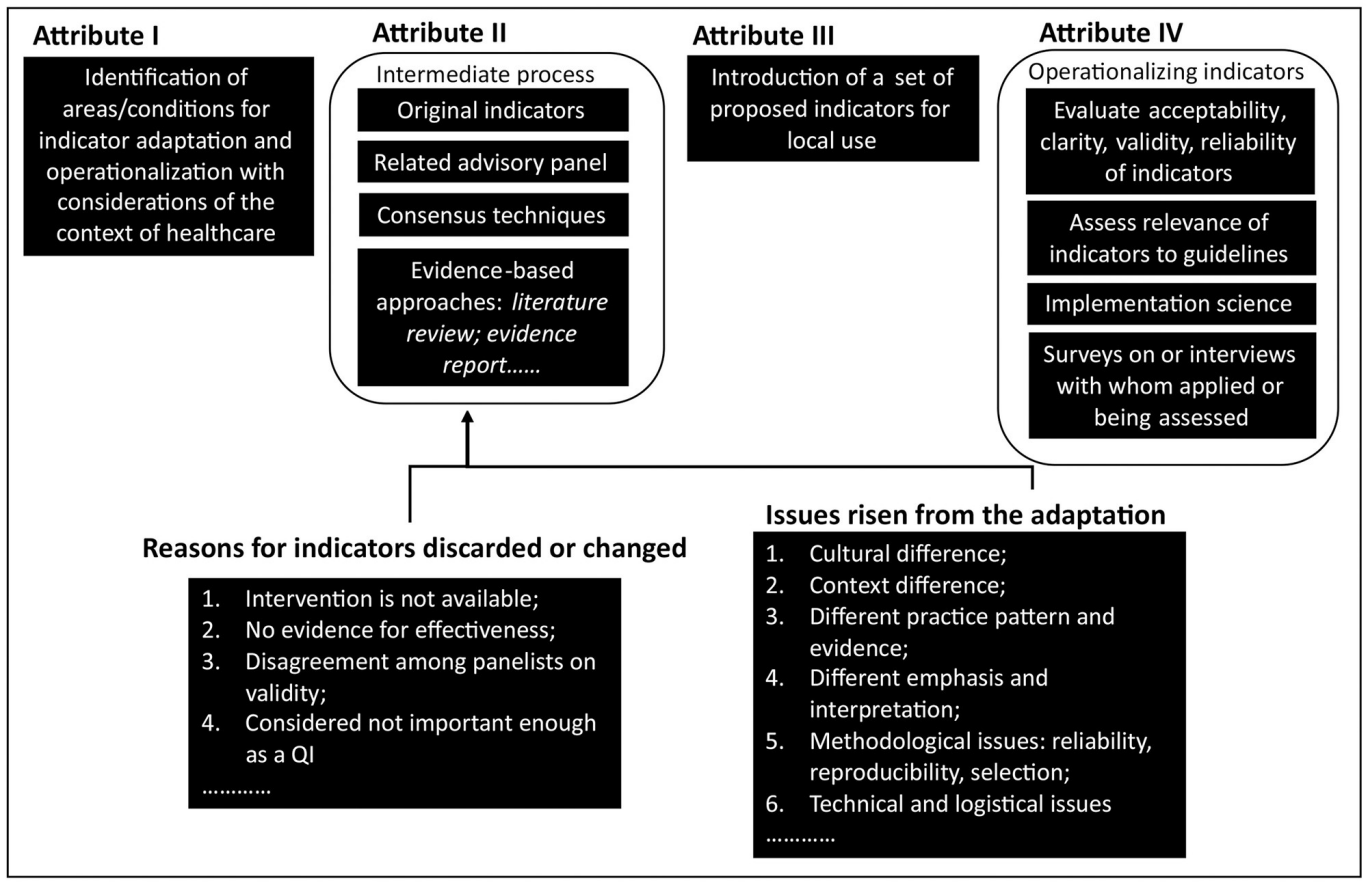
Map of attributes for adaptation process.

The total number of proposed QIs across included studies ranged from 4 to 174, which combined the original set and newly added QIs. Three studies included new QIs in addition to the original set, which accounted for 2 to 21.8% in proposed QIs. After the adaptation process involved in four included studies, at least half of proposed QIs were approved as valid for use, and the range of approval rate for proposed QIs was 56.3 to 85.7%, which indicated that 17 to 76 of QIs were discarded in the process. Of these QIs proposed, 51–98 QIs of the original set were nearly identical without any change, and only 4 QIs were reported to be adapted with major changes [19]. Only two studies [16, 19] reported reasons or provided some comments on why QIs were discarded or changed in the adaptation. Fig 2 maps reasons or comments discarding or changing a QIs in the adaptation.

For the four studies [16, 18, 19, 30], only one study reported that transferability of the existing QIs was feasible, otherwise several issues including cultural context, practice difference, methodological issues, etc., should be considered in the adaptation process. None of these studies formulated a systematic or consistent adaptation approach addressing issues risen from the adaptation process. On the other hand, for six studies that did not adopt an adaptation process, all of them operationalized the existing QIs without any change. Three of the six studies reported that original QIs were easy to apply and acceptable, accounting for over an 80% approval rate for each QI used, and one reported that QIs were operationalized in assessing the quality of care, while two studies found that QIs without adaptation were not fully acceptable. Similar to studies adopting an adaptation process, issues included structure difference, different interpretation of QIs and cross-cultural validation should also be further considered in the adaptation process while operationalizing QIs. Based on steps recommended by Beaton et al. [35] and the proposed methodology used in the latest research [22], none of included studies involved the use of context framework or programme theories (e.g. Consolidated Framework for Implementation Research, Theoretical Domains Framework) to evaluate the adaptation process for local implementation or provide a mechanism for adaptation. Fig 2 maps main issues mentioned in studies that may potentially affect the process of adapting QIs for local use.

We mapped attributes of the adaptation process in Fig 2. A systematic approach of adaptation essentially included four attributes, which were 1) identification of areas/conditions for indicator adaptation and operationalization; 2) intermediate process with combinations and considerations of components listed in Fig 2; 3) introduction of a set of proposed indicators for local use; 4) operationalizing QIs adapted with the aim to evaluate the applicability of them with the implementation methods incorporated if applicable.

## Discussion

The scoping review was conducted to specifically examine evidence adapting or operationalizing an existing set of QIs for local use. Our results highlight the essential role and four attributes of the adaptation process in transferring QIs between countries and different healthcare settings. Four out of ten included studies involved using a consensus process like RAND/UCLA and Delphi for evaluating the acceptability or usability of QIs for local use. The remaining six studies operationalized QIs by involving a multi-disciplinary staff team in medical records to assess validity and acceptability of these measures without an adaptation process. No study included involved using any implementation methods to conduct an assessment on the adaptation process. Most studies with good quality reporting were from European countries and involved QIs developed and implemented within similar settings. However, remarkable variability of how to apply an existed set of QIs locally was observed, relating to the lack of a systematic adaptation approach in adapting and operationalizing the existing QIs, which justified the necessity of the adaptation.

To apply QIs developed in a certain setting or condition, adaptation for the local context and a specific purpose, even within one country, is needed and should be conducted in a systematic manner [4]. Some studies suggested essential steps should be followed in the process of a cross-cultural adaptation, combining different approaches like evidence-based methods, consensus techniques and qualitative or implementation research [22, 35]. Further widespread and well-established method for the development of clinical guidelines should also become an important source to help shape QIs adaptation and dissemination [36]. However, the absence of a standard and systematic adaptation approach process posed challenges in retrieving citations from databases. Therefore, we found that very few studies applied existing QIs, considered as a useful evidence-based quality improvement tool, outside where that were developed. About 62% of the articles were excluded in this study due to the involvement of developing and implementing QIs on their own resources and at high cost [16]. Specifically, the development of several sets of QIs for populations with osteoarthritis were duplicate efforts addressing the assessment of the same process, like weight management recommendations that patients should receive from physicians [37].

Similar to the development process and systematic process of guidelines development, the adaptation process should ideally take place in an iterative way combining inputs from researchers and other stakeholders [38]. Based on what we found in this study, we proposed a systematic approach with four potential attributes should be addressed during the adaptation process. First of all, areas/conditions of interest for QIs adaptation should be targeted by a review of literatures systematically conducted, which is served as an input for the selection. With reference to the adaptation and cross-validation of the US Primary Care Assessment Scales in different countries [22, 39–42], several steps should be taken in the intermediate process, which include but not limited to 1) identification of original set of QIs; 2) an expert panel and user focus group and interviews; 3) consensus techniques (e.g. Delphi process, RAND/UCLA, Nominal Group Technique process); 4) review of QIs wording for local use;. Although the review of scientific evidence is not imperative in the adaptation process, it served to promote mutual understanding across different contexts, and inform the development and adaptation process. After the introduction of a set of proposed QIs for local use, the degree to which QIs have been approved and whether QIs statements are relevant to clinical guidelines need to be assessed by the rigorous method validated in the development process to facilitate decision making [35, 43]. In the assessment, implementation theories, models and frameworks should be used to a deeper understanding of the fundamentals, processes and contexts that shape how adaptation process works and the interactions between different stakeholders [44]. The qualitative content extracted from the process are served to elicit and analyze the impact and value of QIs adaptation and to inform the future implementation [45].

Furthermore, several issues risen from the existing evidence should be taken into account during the intermediate process for the adaptation of QIs. In our study, we found that the approval rate of QIs involved in included studies ranged from 56.3 to 85.7%, and no study could conclude that introducing existing QIs without adaptation was just a matter of copy and paste [4]. Marshall et al. [16] and van der Ploeg et al. [19], adapted US QIs for local use in the UK and Netherlands separately, both indicated that issues like differences in professional practice, expert opinion, panel process, integration of literature source and healthcare context often lead to controversy and inconsistency in the approval of QIs for local use in two countries. For example, they found that the body of evidence reviewed by US researchers differed in content and literature source from that conducted in the UK and Netherlands, for which some QIs were discarded by panellists. In addition, the information infrastructure used to extract data and test the indicator and healthcare systems are different in different regions [4]. Several studies included in this study stated that regional and contextual variations in the structure and process of healthcare systems are major contributors to the failure of directly adopting QIs without any adaptation. In Asia-Pacific countries, more than half of 12 European QIs for dementia were found to be problematic to be used in residential long-term facilities which were different settings for which the QIs were applied [21].

On the other hand, contextual factors should be emphasized at the start of identification of an areas or conditions of interests for QIs adaptation. In the conceptual model of health care, although outcomes are of greatest interest to patients, improving process and how process improves outcomes become the primary target for quality improvement [27, 43, 46]. Process QIs were found in all included studies of this review, supported by findings that the volume of process QIs in The National Quality Measure Clearinghouse increased almost 10 times in a decade between 2003–2013 [27]. Process measurement of healthcare involving QIs has become increasingly common practice and a statutory obligation for administrations worldwide to identify gaps, advance decision-making and inform healthcare policy and delivery [12, 47]. In our study, we found studies in both primary and secondary care settings [20]. Consistent with current knowledge of the development and implementation of QIs, QIs are mostly designed for preventive care and care for chronic conditions, and there is a paucity of QIs for emergency outbreak and infectious diseases [43]. For example, the Coronavirus disease 2019 (Covid-19) pandemic has demonstrated health systems failure in terms of outbreak preparedness and coping with a sudden surge in demand for services such as acute care and rehabilitation service [48]. How to provide patients infected by Covid-19 or not with a consistent level of care poses great challenges on healthcare systems during the pandemic and beyond. Therefore, adaptation of QIs in intensive care units and respiratory medicine and implementation of them in the response to Covid-19 may provide for enormous opportunities to reshape the healthcare delivery system [49, 50].

### Strengths and limitations

The study has several strengths. First, our scoping review was conducted in compliance with the guidance endorsed by the Joanna Briggs Institute [24], and supported by a research librarian with expertise in search strategy development and evidence synthesis. Second, our review serves as a good starting point to provide data on using and adapting existing QIs for local use, in order to inform policy makers, healthcare professionals and other stakeholders in how to introduce a set of QIs in a cost-effective manner. Third, the need for adaptation of existing QIs for local use was identified by the findings of this study, which will inform future research on this topic. Lastly, we worked closely with a multidisciplinary and cross-cultural research team in conducting the review and interpreting the findings.

It is worth noting that some limitations exist in this study. First, the quality of studies may not be appropriately assessed by SQUIRE 2.0 as the checklist is intended for reports that describe system level work and establish observed outcomes due to interventions, although quality assessment of studies is not required for a scoping review. Second, studies published only in English or Chinese were searched in this study by which all relevant studies may not be identified. Third, the lack of a systematic adaptation approach for QIs may have excluded some relevant studies.

## Conclusions

Evidence gaps and future research trends are identified by conducting a scoping review (with at least 10 studies available on a specific topic) like this [51]. In summary, existing QIs serve as a good starting point for introducing a set of QIs for local use, while we found QIs developed in different contexts for different purposes were not adequately adapted before being applied and operationalized in other settings. Adaptation of QIs under a systematic approach is critical for local use, and our review firstly identifies four attributes of a systematic adaptation approach, combined with several issues need to be addressed during the adaptation, to inform future research planning for QIs adaptation. In the future, adaptation of QIs in a systematic manner may potentially provide policy makers, care providers, stakeholders and patients with a new pathway to initiate quality improvement in healthcare and gradually reshape healthcare systems.

## Supporting information

S1 Appendix Search strategies. (DOCX)

S2 Appendix Screening tool. (DOCX)

S3 Appendix PRISMA-ScR checklist. (DOCX)

## Abbreviations

ACOVE: Assessing Care Of Vulnerable Elderly
PROSPERO: International Prospective Registry of Systematic Reviews
PRISMA-ScR: Preferred Reporting Items for Systematic Reviews and Meta-Analyses extension for Scoping Reviews
QIs: Quality Indicators
SQUIRE: Standards for Quality Improvement Reporting Excellence
UCLA: University of California, Los Angeles
UK: United Kingdom
US: United States
