# Supplementary material for: What is the value and impact of the adaptation process on quality indicators for local use? A scoping review
Source: PLoS One. 2022 Dec 8;17(12):e0278379. doi: 10.1371/journal.pone.0278379 (PMC9731415; doi:10.1371/journal.pone.0278379)
Supplement: S1 Appendix — (DOCX) [file pone.0278379.s001.docx]

**Appendix I.** Search strategies

**Medline：**

1. (Developing a framework of, and quality indicators for, general practice management in Europe).m_titl.

2. Quality Indicators, Health Care/

3. ((healthcare or health care) adj3 quality indicator*).mp. [mp=title, abstract, original title, name of substance word, subject heading word, floating sub-heading word, keyword heading word, organism supplementary concept word, protocol supplementary concept word, rare disease supplementary concept word, unique identifier, synonyms]

4. 2 or 3

5. quality assurance, health care/ or benchmarking/ or clinical audit/ or medical audit/ or nursing audit/ or guidelines as topic/ or practice guidelines as topic/ or total quality management/ or quality improvement/

6. (benchmark* or (quality adj3 (assurance or assessment* or improvement or management))).mp. [mp=title, abstract, original title, name of substance word, subject heading word, floating sub-heading word, keyword heading word, organism supplementary concept word, protocol supplementary concept word, rare disease supplementary concept word, unique identifier, synonyms]

7. 5 or 6

8. "Delivery of Health Care"/og, st [Organization & Administration, Standards]

9. Health Services for the Aged/og, st or Primary Health Care/og, st [Organization & Administration, Standards]

10. (healthcare or health care).mp. [mp=title, abstract, original title, name of substance word, subject heading word, floating sub-heading word, keyword heading word, organism supplementary concept word, protocol supplementary concept word, rare disease supplementary concept word, unique identifier, synonyms]

11. 6 and 10

12. 8 or 9 or 11

13. 4 or 7 or 12

14. cross-cultural comparison/ or Consensus/

15. ((transcultural or cross-cultural or cultural) adj3 (comparison* or study or studies)).mp. [mp=title, abstract, original title, name of substance word, subject heading word, floating sub-heading word, keyword heading word, organism supplementary concept word, protocol supplementary concept word, rare disease supplementary concept word, unique identifier, synonyms]

16. 14 or 15 [comparison or transfer]

18. 13 and 16

**Embase：**

1. health care quality/ or benchmarking/ or clinical indicator/

2. ((healthcare or health care) adj3 quality indicator*).mp. [mp=title, abstract, heading word, drug trade name, original title, device manufacturer, drug manufacturer, device trade name, keyword, floating subheading word, candidate term word]

3. 1 or 2

4. clinical audit/ or nursing audit/ or practice guideline/ or total quality management/

5. ((quality adj3 (assurance or assessment* or improvement or management)) or benchmark*).mp. [mp=title, abstract, heading word, drug trade name, original title, device manufacturer, drug manufacturer, device trade name, keyword, floating subheading word, candidate term word]

6. 4 or 5

7. 3 or 6

8. cultural factor/ or international cooperation/ or consensus/

9. ((transcultural or cross-cultural or cultural) adj3 (comparison* or study or studies)).mp. [mp=title, abstract, heading word, drug trade name, original title, device manufacturer, drug manufacturer, device trade name, keyword, floating subheading word, candidate term word]

10. (consensus or international cooperation).mp. [mp=title, abstract, heading word, drug trade name, original title, device manufacturer, drug manufacturer, device trade name, keyword, floating subheading word, candidate term word]

11. 8 or 9 or 10

12. 7 and 11

13. primary health care/ or primary medical care/ or elderly care/ or preventive health service/

14. ((primary or preventive or elder*) adj3 (care or service*)).mp. [mp=title, abstract, heading word, drug trade name, original title, device manufacturer, drug manufacturer, device trade name, keyword, floating subheading word, candidate term word]

15. 13 or 14

16. exp rehabilitation/

17. rehab*.mp.

18. 16 or 17

19. 15 or 18

20. 12 and 19

**CINAHL**

**S1** (MH "Clinical Indicators")

**S2** ((healthcare or healthcare) n3 quality indicator*)

**S3** (MH "Quality Assurance") OR (MH "Quality Improvement") OR (MH "Nursing Audit") OR (MH "Benchmarking")

**S4** (benchmark* or (quality n3 (assurance or assessment* or improvement or management)))

**S5** S1 OR S2

**S6** S3 OR S4

**S7** (MH "Health Care Delivery/ST/AM")

**S8** (MH "Health Services for the Aged/AM/OG/ST")

**S9** (MH "Primary Health Care/AM/ST")

**S10** (healthcare or health care)

**S11** S4 AND S10

**S12** S7 OR S8 OR S9 OR S11

**S13** S5 OR S6 OR S12

**S14** transcultural or crosscultural or cultural or countr* or international or inter-institution*

**S15** (MH "Comparative Studies")

**S16** S14 OR S15

**S17** (MH "Transferability")

**S18** transfer* or adapt*

**S19** S17 OR S18

**S20** S16 OR S19

**S21** S13 AND S20

**S22** S16 AND S21
