# Supplementary material for: What is the value and impact of the adaptation process on quality indicators for local use? A scoping review
Source: PLoS One. 2022 Dec 8;17(12):e0278379. doi: 10.1371/journal.pone.0278379 (PMC9731415; doi:10.1371/journal.pone.0278379)
Supplement: S2 Appendix — (DOCX) [file pone.0278379.s002.docx]

**Appendix II.** Screening tool

**Screening Tool and Eligibility Protocol**

**Primary eligibility criteria**

References excluded if answer No (N) to any of the three criteria.

References kept as an interest article if answer Not Sure (NS) to any of the three criteria without answering any N to the three criteria.

1. Is the article available in English or Chinese? (Y/NS/N)
2. Does the article pertain to use quality indicators as a tool for healthcare quality improvement? (Y/NS/N)
3. Quality indicators for any condition or purpose in any healthcare setting are accepted.
4. Any population that either those being assessed or those undertaking the assessment by using quality indicators is accepted.

**Final eligibility criteria**

References excluded if answer No (N) to any of the three criteria.

References kept as an interest article if answer Not Sure (NS) to any of the three criteria without answering any N to the three criteria.

**Intervention (Phenomenon of Interest):** Pertains to the concept of ‘adaptation process’

1. Does the article involve adapting or operationalizing a set of the existing QIs and try to operationalize or transform them into a practicable set acceptable for the local context? (Y/NS/N)

**Outcomes (Evaluations):**

1. Are outcomes in the article pertaining to the acceptability or usability of quality indicators followed by the adaptation process? (Y/NS/N)

**Reviewer Decision:** Does the article meet the requirements of the above criteria? (Y/NS/N)

**Discrepancy:** Is there discrepancy between the reviewers? (Y/N)

If the answer is yes, a third party will be invited to resolve any dispute between two reviewers.

**Included articles:** Included with all answers to above criteria are yes.
